# The effectiveness of emergency knowledge training of pediatric medical workers based on the knowledge, skills, simulation model: a quasi-experimental study

**DOI:** 10.1186/s12909-022-03267-0

**Published:** 2022-03-29

**Authors:** Yaojia Hu, Bingya Zheng, Lihui Zhu, Shuo Tang, Qi Lu, Qingqing Song, Na Zhang, Yan Zhong

**Affiliations:** 1 Nursing Department, Hunan Children’s Hospital, Changsha, China; 2 The School of Pediatrics, Hengyang Medical School, University of South China Hunan Children’s Hospital, Changsha, China; 3 Medical Department Emergency Office, Hunan Children’s Hospital, Changsha, China; 4 Department of Cardiology, Hunan Children’s Hospital, Changsha, China; 5 School of Nursing, Hunan University of Chinese Medicine, Changsha, China; 6 Child Health Care Center, Hunan Children’s Hospital, Changsha, China

**Keywords:** Emergency, Simulation, Training, Pediatricians, Basic life support

## Abstract

**Background:**

Basic life support and advanced life support are essential emergency management skills for medical workers, and pediatricians' first aid skills can be improved through emergency knowledge training.

**Methods:**

A controlled pre–post-intervention quasi-experimental study design was used. The study setting was a tertiary children's hospital in China. In November 2019, a KSS model of emergency knowledge learning was developed and tested, and pediatric medical workers (*N* = 1448) were trained with it. The outcome measures were based on an emergency knowledge questionnaire devised by the authors that measured the effectiveness of training by comparing the pre-and post-training scores of the particpants.

**Results:**

Pediatric medical workers scored significantly higher in total emergency knowledge after the training course than before [75.00 (62.50, 85.00) versus 100.00 (95.00, 100.00); *P* = 0.00]. Basic life support and advanced life support knowledge score significantly improved after training. Teamwork scores were significantly higher after the training than before [5.00 (5.00, 10.00) versus 10.00 (10.00, 10.00); *P* = 0.00]. Scores were significantly higher after the training (*P* < 0.001), especially for case analysis questions (*P* = 0.00). The attitudes of the medical workers towards the training were all positive and affirmative.

**Conclusion:**

The KSS model was shown to be effective in improving the emergency knowledge of pediatric medical workers. Future research will be to explore the effectiveness of the model with different participants and at other hospitals or other institutions such as schools, encouraging more people to participate in and evaluate the model to promote its optimization.

**Trial registration:**

Hunan Children’s Hospital, HCHLL-2018-03.

**Supplementary Information:**

The online version contains supplementary material available at 10.1186/s12909-022-03267-0.

## Background

Basic life support (BLS) and advanced life support (ALS) have been recognized as the standard of competence for managing patients in cardiac arrest [1]. High-quality cardiopulmonary resuscitation (CPR) and defibrillation, if indicated, are the central interventions in the initial resuscitation from sudden cardiac arrest (SCA) [2]. Currently, the median hospital survival rate after pediatric in-hospital cardiac arrest is 36%, while the median survival rate for out-of-hospital cardiac arrest is less than 10% [3]. If appropriate resuscitation were administered quickly, tens of thousands of pediatric deaths could be prevented. Compared with adults, children have anatomical, physiological, and behavioral differences that place additional demands on pediatricians in critical situations [4]. Pediatrics residents are graduating with poor self-perceived competency in emergency skills such as bag-mask ventilation and foreign body removal [5]. Whereas pediatricians may have difficulty dealing with arrhythmias, CPR and trauma, junior nurses face the thorny issue of how to perform neonatal CPR, possibly due to a lack of relevant clinical experience [6, 7]. The low level of first aid knowledge among pediatric health care providers explains the rise of emergency literacy training. Providers with limited knowledge and experience handle most pediatric emergencies, and the first aid skills and knowledge of pediatric medical workers are generally low [8]. Poor outcomes in the EMS systems of developing countries are often due to a lack of resources, insufficient training, and other system deficiencies. Despite this, some countries have slowly noticed the importance of developing first aid personnel, focused on optimizing the education and training of workers for initial patient stabilization and resuscitation [9].

Traditional resuscitation courses are usually taught in an intensive 1–2 day format, typically combining theoretical lectures with multimedia presentations, with limited retention of knowledge and skills by medical workers for several months after completion of the resuscitation course [10]. Several studies have pointed out that simulation techniques are effective in developing pediatric clinical skills, that experiential learning through simulation technology students to provide feedback and analysis of clinical emergencies [11–13], and short, high-frequency simulation training sessions help improve CPR skills performance [14]. Such as improved critical action completion, clinical knowledge, psychomotor skill application, and decreased time to CPR and defibrillation [15].

### The KSS model framework

Skill simulation relates to key measures in improving quality of life and the gradual acquisition of cardiopulmonary resuscitation skills during pediatric training [16], and it is an effective method for teaching non-technical and behavioral skills such as teamwork, leadership, communication, and clear roles [17]. Educating on cooperation and working in teams applies to all health professionals and can be adapted to various situations, with a beneficial impact on the emergency skill ability of health professionals working in teams daily [18]. In the past, simulation was often integrated in a fragmented way into training courses, with a lack of comprehensive application. But our KSS model avoids this problem. The knowledge, skills, simulation (KSS) model is similar to simulation-based medical training (SBMT) and has been defined as “the artificial representation of a complex real-world process with sufficient fidelity” [19]. In the training, we implement long-term centralized scenario simulation, establish theoretical schemata through preliminary knowledge training, and finally consolidate the effect through face-to-face skill practice. Our education model is step-by-step, continuously deepening the trainees' skills and knowledge, thus solving the problem of loss of knowledge due to fragmented training time or courses.

### Aim

This study aimed to develop a model for emergency training and to evaluate its effectiveness among pediatric workers. Specifically, our study was to compare the acquisition and improvement and reinforcement of first aid skills among pediatric medical workers. The acquisition and maintenance of first aid skills, especially the level of knowledge and teamwork, was achieved through knowledge, simulation and skill models.

## Methods

### Study design

**Fig. 1:**
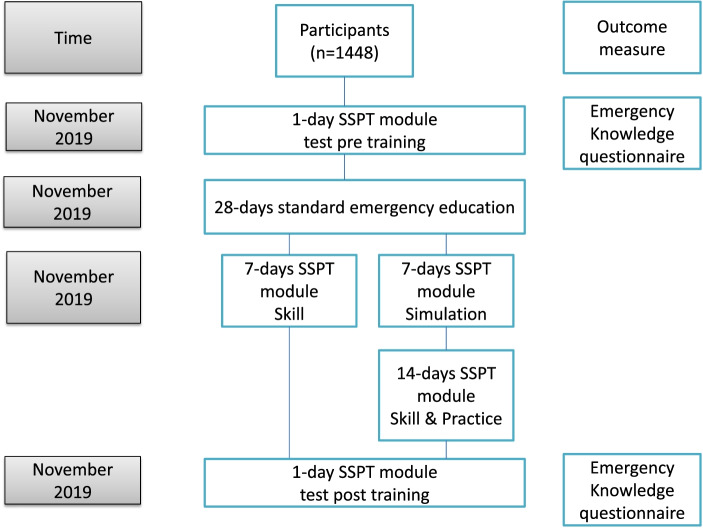
Study outline

The study was quasi-experimental and used a pre–post-intervention designed to assess the efficacy of emergency knowledge training in pediatric workers using the knowledge, skills, simulation (KSS) model (Fig. 1). The research KSS was carried out in strict accordance with emergency training specifications. The study was organized by the hospital emergency department, and ran from 5 November to 5 December 2019.

### Setting

The study was conducted at Hunan Children's Hospital in Changsha, China, a national specialized hospital with 1800 beds, 35 clinical departments, and 9 medical technology departments covering all pediatric specialties. The severe pediatric disease is a national key clinical specialty and the pediatric emergency medical laboratory is a key laboratory in Hunan Province. The research participants were hospital medical workers. Qualified teachers were abundant and teaching conditions good, with 7 emergency medical experts, more than 60 emergency medical experimental researchers, and plentiful emergency equipment.

### Participants

The study included 1448 employees (including nurses, doctors, and technicians) of Hunan Children's Hospital. To ensure the objective authenticity of the study, we used a stratified random sampling method to distribute a two-step questionnaire to the participating hospital pediatric medical workers, emphasizing that their responses were not related to salary or perceptions of their performance. After the employee signed the informed consent form approved by the emergency response department, the participants answered the questionnaire, which was on emergency skills and knowledge. After skill simulation practice and one-to-one training using the new model, the participants answered the same questionnaire again to assess their capacity for emergency care.

### Educational interventions

The duration of the emergency training course based on the KSS model was 28 days, and included on-site safety, patient assessment, rescue and recovery, personal first aid awareness, personal technology, team cooperation, team commander's ability, and condition communication ability. The content of the KSS model was designed for pediatric clinical workers. The model was developed by eight emergency medical experts and three nursing experts who specialized in emergency knowledge and skills, teaching methods, scenario simulation, and emergency communication. The training program for the model was designed by two of the authors (YJH and LHZ), based on the available literature in the field [20–22]. After consulting senior experts in pediatric emergency medicine, BLS education, and hospital training management, the draft content of the model was discussed and revised. Through consultation with experts and developers, the content of the KSS model was finally determined.

The KSS model includes three components: knowledge, skills, and simulation (Table 1). participants began with the knowledge part, after the pre-training test. The skill phase included online lectures over 7 days. The knowledge courses were presented on the internal website of the hospital, with a first aid knowledge question bank and video teaching based on case analysis. participants were free to study independently using the website in their own time. The simulation stage mainly comprised 7 days of team training. The course material was instructed using the demonstration to the whole hospital with case simulations, with a “practice-while-watching” format. Team training not only simulates the process of one person calling for help and one person performing cardiopulmonary resuscitation with a three-person team resuscitation after medical workers find the patient's condition changed in the ward. It also stimulates the overall process of the nurse communicating the condition with the family members and transferring the patient to another department First aid awareness, personal technology, team cooperation, team leadership, and communication ability in the recovery process were comprehensively assessed and trained, and precautions in the rescue process were emphasized again after the assessment. In the skill phase, each department participated in training, helping familiarize other medical workers with first aid procedures through one-on-one guidance by the departments’ trainers, and striving for every medical worker to master the required skills. Proficiency in first aid skills cannot only rely on one-on-one training, but is also achieved by each worker practicing carefully after learning first aid procedures, reducing the time that procedures take by repeated practice. This enhances patient survival by improving the rate and efficiency of first aid resuscitation. During the 14-day practice period, participants ' operational skills were continuously strengthened, their ability to deal with emergency events increased, and tacit cooperation among colleagues improved. Following the three stages of skills, simulation, and practice, all participants were tested. The hospital then organized a training summary meeting to analyze the problems and deficiencies of participants.

**Table 1:** Knowledge, skill, simulation model

Component	Context	Time
Skill	On-site safety, patient assessment, rescue and recovery, personal first-aid awareness, personal technology, team cooperation, team commander's ability and condition communication ability	7-days
Simulation	The delivery format of the course instruction is an operation demonstration of the whole hospital under the case simulation that is followed by ‘practice-while-watching’. Participants were required to engage with a different scenario and role play in clinical emergency settings	7-days
Skill	Participants will receive experienced teacher guidance and one-on-one training and will practice first aid in different situations	14-days

The KSS training supervision system is divided into three levels: hospital level, section level and individual level. The knowledge stage is mainly based on individual self-supervision coordinated with hospital and section level supervision, and the actual contact time for this stage is 2 h of self-help learning after work; the simulation stage is a unified hospital training for 7 days, and all participants attend team simulation training in batches, which is supervised by the hospital-level emergency skills upgrading team; in the skills stage, the department according to the emergency skills upgrading program, the department director and nurse chief emphasized the importance of skills upgrading in the department's morning meeting every day, while the department's operation lead teacher provided further training to the department's staff, and the director and nurse chief supervised during the practice.

This model is also known as “four 7-days”. The first 7 days occurred in November 2019 and included the knowledge component of the model. The second 7 days, comprising the simulation training, was also carried out in November 2019. The final 14 days comprised the skill component of the model, ending in December 2019. All of the above constitute the "28 days" of the intervention.

### Assessment instruments

We used a self-designed questionnaire to assess the effectiveness of participants ' training. The questionnaire was divided into two main parts. The first part consisted of 10 demographic questions (including gender, age, specialty, department, position or titles, educational background, and previous training in CPR). The second part was on first aid knowledge and was based on the 2019 International Liaison Committee on Resuscitation’s International Consensus on Cardiopulmonary Resuscitation and Emergency Cardiovascular Care Science with Treatment Recommendations (ILCOR) [21]. It included 13 single-choice questions (SCQs) in emergency knowledge and an emergency analysis case with 8 SCQs. The scores of these two parts are added together to obtain a total score of 100 for the questionnaire, with a passing score of 60 and a failing score of less than 60. Details of the questionnaire can be found in Additional file 1. Three questions were set for the attitude aspect to explore the attitudes of the pediatric medical workers on completion of the training.

The reliability and validity of the questionnaire were investigated by eight BLS and three ALS teachers from the clinical skills training center of Hunan Children's Hospital, and five CPR experts from the hospital were invited to evaluate the questionnaire content. The questionnaire was randomly distributed to 100 medical professionals in the hospital during the trial phase using a stratified random sampling method. The validity, internal consistency, and reliability of the instruments used in this study were achieved by conducting quasi-experiments to explore the retest reliability using the same questionnaire with repeated measures on the same subjects at different times, and by using factor analysis for each question. Using the Statistical Package for Social Sciences (SPSS version 24.0, IBM Corporation, Armonk, NY, USA), the internal consistency of the questionnaire was calculated to be 0.833 and the retest reliability was 0.899. In addition to this, the results of the factor analysis showed that the KMO value = 0.889, which is close to 1, implying that the correlation between the variables is strong and the more suitable the original variables are for factor analysis, the more influential The factors of the questionnaire scores were early identification, basic life support, and a multidimensional structure. The questionnaire was subjected to review and approval by an evaluation committee and an ethics committee, both at Hunan Children’s Hospital. The reliability of the instrument was assessed by analyzing the closed-ended questions using the Kuder-Richardson 20 (KR20) formula with each subset of questions. The internal consistency of the questionnaire was tested by Cronbach's alpha coefficient.

### Data collection

**Fig. 2:**
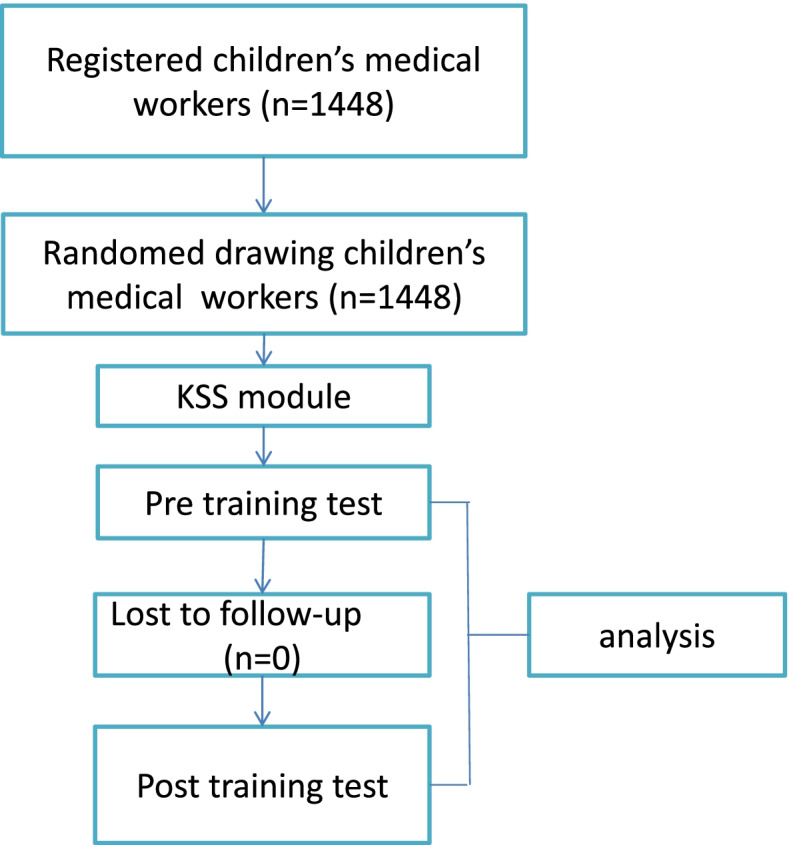
Study flowchart

The 1448 participants received and completed the demographic questionnaire and a test on managing emergencies before the training, and the test was conducted once more after standard emergency education and education using the KSS model (Fig. 2). The questionnaires were anonymous, with no recognizable mark or information. The analysis was performed by an author (YJH) who does not participate in the organization of staff assignments or training models. During the clinical emergency skills training, all personnel had to complete a team cooperation emergency scene simulation and one week of independent emergency knowledge learning. Otherwise, data collection and results may have been affected.

### Statistical analysis

For descriptive statistics, quartiles and medians were used. Descriptive statistics were used for the attitudes of the medical workers to derive the frequency of each outcome. Nonparametric data based on different characteristics, such as gender, age, and title were analyzed using the Wilcoxon rank-sum test. Chi-square test was used for enumeration numerical data, with the effect of training evaluated by comparing the participant scores before and after training, for example, a comparison of the number of qualified people for each of these items. We considered *P* < 0.05 to be statistically significant.

## Results

### Participants’ characteristics

A total of 1448 pediatric workers participated in the research. The majority of participants were women (*n* = 1220; 84%), and the majority of medical workers had junior professional titles (*n* = 808), accounting for 56% of the total. Most of them (51%) were 31–40 years old. Of the participants, 98% had received CPR training in the past. Participants mainly included nurses and undergraduate students, accounting for more than 60% (Table 2). There were no differences in test scores before training by gender, occupation, age, title, education background, and previous CPR training, with a (*P* > 0.05) median score across these subgroups of about 75 points (Table 3).

**Table 2:** Demographic characteristics of parricipants

Variable	N (%)
Total participants	1448 (100%)
Gender	
Men	228(16%)
Women	1220 (84%)
Age (in years)	
≤ 30y	506(35%)
31-40y	734(51%)
41-50y	146(10%)
≥ 51y	62(4%)
Title	
Junior	808(56%)
Intermediate	396(27%)
Deputy senior	181(13%)
Senior	63(4%)
CPR training	
Already participated	1422(98%)
Not participated	26(2%)
Qualification	
Associate degree	126(9%)
Bachelor's degree	968(67%)
Master's degree or above	354(24%)
Profession	
Doctor	395(27%)
Nurse	896(62%)
Technician	157(11%)

**Table 3:** Comparison of CPR scores by different characteristics – before training

**Variable**		**Score *M (P***_***25***_***,P***_***75***_***)***	***Z***	***p***
**Gender**	Male	75.00 (62.50,84.38)	-0.065	0.95
	Female	75.00 (62.50,85.00)		
**Age**	≤ 30 g	75.00 (65.00,82.50)	3.818	0.282
	31-40 g	72.50(60.00,85.00)		
	41-50 g	72.50(60.00,85.00)		
	≥ 51 g	75.00 (63.75,85.00)		
	Junior	75.00 (62.50,82.50)	6.311	0.097
**Title**	Intermediate	72.50(60.00,82.50)		
	Deputy senior	77.50 (62.50,90.00)		
	Senior	72.50 (65.00,85.00)		
**CPR training**	Already participated	75.00 (62.5,85.00)	-1.243	0.214
	Not participated	75.00 (55.00,80.00)		
**Qualification**	Associate degree	75.00 (60.00,85.00)	0.351	0.839
	Bachelor's degree	75.00 (62.5,84.38)		
	Master's degree	75.00 (62.50,85.00)		
**Profession**	Doctor	75.00 (65.00,85.00)	1.681	0.431
	Nurse	75.00 (62.50,85.00)		
	Technician	72.50(60.00,82.50)		

*P* < 0.05 is statistically significant

### Differences before and after training

There were significant improvements in the results for each question in the knowledge test after the training. Table 4 shows the comparison of scoring of each question before and after the training. Except for questions 3, 6, and 10, the proportion of participants who answered the questions correctly increased significantly after the training, to about 95% of the total participants (*P* = 0.00). It was obvious that the participants knew first aid after the training. Frequencies of correct answers in the pre-and post-training tests are shown in Table 5. The number of participants answering correctly the eight questions based on a case analysis of emergency treatment knowledge increased significantly after the training (*P* = 0.00).

**Table 4:** Comparison of each question answered correctly before and after the training

Component	Pre-training		Post-training		*X*^*2*^	*P*
	Correct number (*n*)	Correct rate (%)	Correct number (*n*)	Correct rate (%)		
**Question1**	1143	78.90	1347	93.00	119.21	0.00*
**Question2**	539	37.20	1214	83.80	658.53	0.00*
**Question3**	1372	94.80	1431	98.80	38.67	0.00*
**Question4**	1242	85.80	1417	97.90	140.73	0.00*
**Question5**	810	55.90	1323	91.40	468.29	0.00*
**Question6**	1213	83.80	1372	94.80	91.06	0.00*
**Question7**	859	59.30	1378	95.20	529.15	0.00*
**Question8**	924	63.80	1376	95.00	431.62	0.00*
**Question9**	1107	76.50	1379	95.20	210.20	0.00*
**Question10**	1337	92.30	1412	97.50	40.31	0.00*
**Question11**	964	66.60	1373	94.80	370.83	0.00*
**Question12**	954	65.90	1376	95.00	391.06	0.00*
**Question13**	762	52.60	1367	94.40	649.14	0.00*

^*^P < 0.01 is statistically significant

**Table 5:** Comparison of the accuracy of case analysis questions before and after training

Component	Pre-training		Post-training		*X*^*2*^	*P*
	Correct number (*n*)	Correct rate (%)	Correct number (*n*)	Correct rate (%)		
Question14-(1)	1382	95.40	1440	99.43	46.65	0.00*
Question14-(2)	701	48.40	1334	92.10	662.28	0.00*
Question14-(3)	1276	88.10	1369	94.50	37.73	0.00*
Question14-(4)	1373	94.80	1439	99.40	53.41	0.00*
Question14-(5)	1361	94.00	1430	98.80	47.05	0.00*
Question14-(6)	479	33.10	1236	85.40	950.54	0.00*
Question14-(7)	1258	86.90	1410	97.40	109.99	0.00*
Question14-(8)	816	56.40	1354	93.50	532.07	0.00*

*P < 0.01 is statistically significant

Table 6 compares the scores for BLS, ALS, and other variables before and after the training, and shows an overall improvement in median score to between 75.00 (62.50, 85.00) and 100.00 (95.00, 100.00). By comparison, the BLS score improved to 60.00 (55.00, 60.00) after training, compared with 45.00 (35.00, 55.00) before training, an improvement of 15 points (*P* = 0.00). ALS and team communication scores also improved to some extent (*P* = 0.00). Using KSS-based training evidently significantly improved the knowledge of all participants. ( BLS/ALS scores are counted from the corresponding entries.)

**Table 6:** Differences in knowledge scores of all sections before and after training

**Variable**	**Score M (P25, P75)**		*Z*	*P*
	**Pre-training**	**Post-training**		
**BLS score**	45.00(35.00, 55.00)	60.00(55.00, 60.00)	-27.14	0.00*
**ALS score**	20.00(15.00, 20.00)	25.00(25.00, 25.00)	-25.83	0.00*
**Team communication score**	5.00(5.00,10.00)	10.00(10.00, 10.00)	-22.34	0.00*
**Overall score**	75.00(62.50, 85.00)	100.00(95.00,100.00)	-29.57	0.00*

^*^P < 0.01 is statistically significant

There were a total of 41 departments in the hospital, each representing a team, and they all showed a significant improvement in their scores after the training completion. The differences between the different departments did not vary much from the expected results. Before the training, the median total score fluctuated between the different departments in the range of 63.75 to 75, and after completing the training, the scores between the different departments improved to the range of 90 to 100.

Table 7 shows the attitudes of the medical workers toward the training. 98.59% of the medical workers accepted the training approach, 89.75% of the medical workers thought the current training exercise content was adequate, but more than 400 people still suggested adding other exercise feedback content; the vast majority were willing to participate in the follow-up training, as detailed in the table.

**Table 7:** Medical workers' attitude towards knowledge related to emergency skills post-training

Projects	Number of people n	Proportion %
**Do you approve of our KSS training model?**		
Acceptance of the current approach to medical emergency drills	1428	98.59%
The content of the current training exercises is sufficiently complete (participants, exercise scenarios, exercise process, exercise effects)	1299	89.75%
Suggestions for additional walkthrough feedback	430	29.69%
**What other emergency skills knowledge training would you like to participate in in the future?**		
Medical emergency safety management knowledge training	1314	90.74%
Medical emergency laws and regulations knowledge training	1234	85.21%
Medical emergency clinical expertise training	1285	88.71%
Medical Emergency Skills Training	1308	90.37%
**Which of the following training methods would you choose?**		
Self-financed	726	50.12%
Half self-pay + half hospital reimbursement	1337	92.31%
Hospital Reimbursement	1448	100%

## Discussion

### Summary of main findings

The on-site assessment is the first step in preparing for first aid, which is emphasized many times even in adult CPR [23]. Seventy percent of the medical workers had a pretty decent grasp of site assessment skills before the training. We found that in addition to an increase in the frequency of single correct answers, scores in BLS and ALS knowledge increased significantly post-training, and team performance improved. Of significant interest is that only 52.6% of participants knew how to properly defibrillate after a cardiac arrest before the training, and after the training, the accuracy rate increased to 95.4%.In particular, participants ' approaches to CPR programs, including victim assessment, initiation of CPR, and collaborative team communication, were improved following the four-step first aid training approach, which demonstrates that our training is effective. The total score before training was unsatisfactory even for experienced medical workers, with most of them only scoring 75 points, which is at the pass level (a score greater than or equal to 60 points indicates a pass), and insufficient to be considered an excellent standard (Table 3). This suggests it is equally important for experienced medical workers to attend emergency training regularly [24]. In the case of an emergency, the doctor leads the actions of the whole team [24]. And this was so in our case study in which the pediatrician took the initial lead, raising questions such as "you and the nurse are on emergency duty together, what should you do as the commander of the resuscitation? Direct nurses, to manage patient breathing? Can the nurses use resuscitation bags to establish breathing?" to test their leadership, emergency response, and communication skills. Based on the KSS model, all the abilities of the medical workers were improved. The vast majority of participants had a positive attitude toward the training and showed a strong initiative for relearning and retraining, which laid the foundation for us to continue related research in the future.

### Comparison with existing literature

These results are by those of a previous study evaluating university students in the health field, which reported that 96% of participants achieved CPR performance scores > 70% [25]. Studies have shown that continuous days or weeks of training are beneficial for students learning CPR, and the less time between exercises, the less likely they are to make operational mistakes and the more likely they are to improve their skills [26]. The KSS training model trained the participants for four consecutive weeks. After their knowledge and skills were strengthened, clinical case simulation training was carried out while knowledge was still fresh in their minds. Our study showing the benefits of developing and maintaining CPR skills with immediate practice and shorter times between training sessions are broadly consistent with other research results [26].

Performing immediate CPR and early defibrillation requires awareness of the emergency and an early call for help [27]. We set up questions related to calling for help from the staff's own first aid awareness, asked "the team leader asked you to provide mask ventilation during the resuscitation attempt, but your skills are not perfect. What appropriate actions should be taken to recognize your limitations?", encourages them to seek help from their teammates in time when they are unable to solve a problem. There seems to be less research on such similar studies, and the general focus is on the frequency, depth, and duration of compression [28].

In the current study, a significant knowledge deficiency was noted in the correct steps in operating an AED. It is well accepted that hands-on training is effective for reinforcing the quality of CPR. A focus on chest compression and AED skills training made this training program more effective [29]. Repeating training can maintain emergency skills for longer [30]. In the study by Nimbalkar et al. [31], negative results were found after 3 months of training with a total learning time of 18 h over 3 days. In contrast, Rubio-Gurung et al. [32] tracked participants after 1 month of continuous study (4 h per day) and found that simulation training was effective in improving first aid skills and teamwork behaviors with long-term positive effects. One week of situational simulation training and two weeks of practice training has a positive impact on the consolidation and maintenance of knowledge in BLS, AED, and ALS, showing that frequent refreshers at short intervals improve not only skills but also confidence. The results of this study are consistent with those of other researchers [33, 34]. The effects of using a knowledge simulation model cannot be ignored, and the results suggest that in future research on emergency knowledge training, training of knowledge and skills is an indispensable component. If emergency knowledge and skills are not frequently used or practiced, they are likely to be gradually forgotten over time, so frequent intensive training is necessary. First aid knowledge and skills are essential to medical work, and if our hospital conducts training every year, we can effectively maintain the first aid knowledge of medical workers and stability in their ability to perform first aid procedures, with greater homogeneity. Training time and duration are critical points for long-term retention of improved skills.

Feedback is an important factor in tracking the effectiveness of the training. In addition to feedback on performance, it is also important to focus on the respondents' attitudes towards the study and what they think about the training because the researcher can experience what it is like to be in each phase of the training [35]. Very glad that our subjects were happy with this type of training and were very motivated to learn, hoping to have training on relevant legal knowledge, skills knowledge, and safety knowledge, and about 50% were willing to go for CPR training even if they were studying at their own expense, indicating that they were aware of the importance of first aid knowledge. We believe that the deeper the learners are immersed in the training environment, the more their behavior will reflect what they have learned and practiced.

### Methodological challenges

Our strengths are more obvious. While this model has been similar internationally, there is no short-term, staged, large sample of training, and even when there is short-term training, it is very focused on filler learning with small sample size. Short-term, high-frequency training helps to improve CPR skills, and our intervention model is phased, with each phase providing further reinforcement of knowledge and the practice phase consolidating the effects of memory through continuous recall. This is what sets our study apart from other studies. Our educational model is progressive, continuously deepening the skills and knowledge of the trainees, thus solving the problem of knowledge loss due to scattered training time or courses. Our training is of scale, and this wave of all medical workers learning emergency knowledge not only drives the learning atmosphere in this section but also allows more people to learn about first aid.

Relying on questionnaire testing alone, our data are inherently limited. The same questionnaire set was used for the pre-and post-training tests have some limitations, after the first test, the study subjects may go to actively search for the correct answers and get high scores in the second test, but some of the questions were not mastered. It is possible that the participant may have a lack of additional knowledge that is not detected by us. Due to the voluntary nature of this study, there may be a selection bias (volunteer effect), as respondents interested in this type of training may be more inclined to participate. Nevertheless, we believe that our results provide valuable insights.

### Implications for educators and future research

For EMS training education, our study provides new ideas and systematic thinking for training programs, but there is an equal need to track training effectiveness and clinical response outcomes over time. We included a considerable number of respondents, all of whom were drawn from hospital professionals, fully representing all expected subgroups within the hospital. Our study contributes to the field of simulation education, skills training, and guides future training research and a systematic educational mindset based on knowledge, skills, and simulation for those who play an important role in developing programs, policies, or procedures in clinical practice. For prospective consideration, future studies could refine the protocol of the study through randomized study subgroups to drive new advances in the field of education. Pediatricians have little exposure to emergencies during their training, leaving them unprepared for life-threatening situations because children rarely require resuscitation [36]. Medical students and nursing staff receive first aid knowledge in hospitals and universities that is often administered to adults and have limited exposure to pediatric emergencies unless they attend specialized pediatric academic conferences [37]. Moreover, there has been a large gap between pediatricians and pediatric practitioners; therefore, we suggest that pediatric emergency training courses could be included in future institutional education to allow more people to understand pediatric emergencies and join the pediatric practice.

## Conclusion

This study demonstrates the effectiveness of the KSS teaching model used by Hunan Children’s Hospital for emergency skill training. This intensive training based on knowledge and skills simulation testing will help medical workers to provide high-quality first aid knowledge training in a short time, and generally improve the skill, communication, and knowledge levels of medical workers. The attitudes of the medical workers towards the training were all positive and affirmative, which is an indication of the success of our training. Hospitals can provide real-time feedback and improvements according to the 2019 ILCOR recommendations and may consider incorporating the concept of the KSS training model into emergency training at adult general hospitals or the CPR training plan of medical schools.

## Supplementary Information

**Additional file 1.** Emergency knowledge assessment questionnaire.

## Data Availability

All data generated or analyzed during this study are included in this article and its supplementary information files.

## Abbreviations

BLS: Basic life support
CPR: Cardiopulmonary resuscitation
ALS: Advanced life support
AED: Automated external defibrillator
KSS: Knowledge, skills, simulation
SBMT: Simulation-based medical training
ACLS: Advanced cardiac life support
SCQs: Single-choice questions
