# Supplementary material for: The effectiveness of emergency knowledge training of pediatric medical workers based on the knowledge, skills, simulation model: a quasi-experimental study
Source: BMC Med Educ. 2022 Mar 29;22:213. doi: 10.1186/s12909-022-03267-0 (PMC8966279; doi:10.1186/s12909-022-03267-0)
Supplement: Supplementary file 1 — Additional file 1. Emergency knowledge assessment questionnaire. [file 12909_2022_3267_MOESM1_ESM.docx]

**Emergency knowledge assessment questionnaire**

**Part I: General Information**

**1. Your gender:**

A. male

B. Female

**2. Your final education:**

A. Junior College

B. University

C. Master’s degree and above

**3. Your identity:**

A. Doctor

B. Nurse

C. Technician

**4. Your work number:**

**5. Your department:**

**6. Your age:**

A. Under 30y

B. 31y-40y

C.41y-50y

D. Above 50y

**7. Professional rank:**

A. Primary title

B. Middle title

C. Vice-senior title

D. Senior title

**8. Executive function:**

A. Vice-section chief

B. Section chief

C. Other

D. N/A

**9. Have you attended in CPR training:**

Yes:

A. In school

B. In the hospital

C. Outside the hospital

D. No

**PartⅡ: Knowledge about emergency skills**

**1.** You saw an 8-year-old boy fall and was the first rescuer to arrive at the scene. You found that he was lying motionless on the ground. What was the first measure you took in this case?

**2.** You saw an 8-year-old boy who fell and was the first rescuer to arrive at the scene. You found that he was lying motionless on the ground. When you touched his shoulder and shouted, "Are you okay?" He did not respond, what is your best next step?

**3.** What is the chest compression breathing rate when providing adult CPR?

**4.** The speed and depth of chest compressions in adult patients?

**5.** You are performing CPR with your bare hands. What measures should you take

when more rescuers arrive?

**6.** If you suspect a headless patient with a head or neck injury, what is the preferred method to open the airway?

**7.** The team leader asked you to perform mask ventilation during the resuscitation attempt, but your skills are incomplete. What appropriate actions should be taken to recognize your limitations?

**8.** When the team leader assigns your tasks, what appropriate behaviors can demonstrate closed-loop communication?

**9.** What is the correct compression-ventilation ratio of a single rescuer for a 3-year-old child?

**10.** If there are 2 or more rescuers, how many years of patients are recommended to

use double thumbs?

**11.** What is the correct depth of chest compressions for children?

**12.** What is the correct depth of baby chest compressions?

**13.** What should emergency rescuers do after shock defibrillation during CPR?

**14.** You are a pediatric on-duty physician (or rescue commander). You suddenly heard the call of the nurse on duty. The inpatient NO.1 bed suddenly stopped breathing. You immediately rushed to the 01 bed room. The nurse has started chest compressions.

(1) At this time, you should

A. Call your superior doctor

B. Establish venous access

C. Give oxygen

D. Call the second-line doctor or emergency team by phone, and rescue immediately, and cooperate with the nurses to do two people recovery.

(2) The ratio of chest compression frequency to breathing at this time is

A.30: 2

B.3: 1

C.15: 2

D.10: 1

(3) You rescue with the nurse on duty, you should

A. As the rescue commander

B. Command the nurse to manage breathing

C. Nurses can use resuscitation capsules to establish breathing

D. all of the above are correct

(4) Pay attention when using the resuscitation bag

A. EC method

B. Regardless of mask size

C. chest expansion no more than 1 second

D. No need to open the pressure reducing valve

(5) After 10 cycles of CPR with the nurse, you should evaluate

A. Heart rate

B. Breathing

C. Carotid pulse

D. All of the above are correct

(6) At this time, the heart rate gradually recovered, but the heart sound was dull, and the heart rate was 40 beats / min. The following processing errors are

A. CPR

B. Give epinephrine 0.1-0.2mg / kg, intravenously

C. Epinephrine is diluted to 1: 10000

D. 1: 10000 epinephrine 0.1-0.2ml / kg given intravenously

(7) After using the medicine, evaluate again

A. Breathing

B. Heart rate

C. Aortic pulsation

D. All of the above are correct

(8) At this time, the heart rate cannot be heard, and the ECG monitor shows a ventricular fibrillation. At this time, the following processing errors are:

A. Prepare for defibrillation

B. Defibrillation energy is selected according to 2-4J / kg

C. Stop chest compressions and positive pressure ventilation during defibrillation preparation

D. 10 cycles of cardiopulmonary resuscitation immediately after defibrillation
